# Cardiovascular Diseases in Portuguese: The Importance of Preventive Medicine

**DOI:** 10.5935/abc.20180103

**Published:** 2018-07

**Authors:** Fausto J. Pinto

**Affiliations:** Clínica Universitária de Cardiologia, CAML, CCUL, Universidade de Lisboa, Lisboa - Portugal

**Keywords:** Cardiovascular Diseases / mortality, Cardiovascular Diseases / prevention & control, Morbidity, Risk Factors, Myocardial Ischemia, Myocardial Ischemia, Terapeutics / trends, Community of Portuguese- Speaking Countries

Cardiovascular diseases (CVD) are the leading cause of mortality and morbidity worldwide.^1^ Because several CVD have sequelae that significantly impact the life of affected individuals, knowing the importance of those diseases, as well as their associated factors, is essential to develop preventive measures to reduce that impact.^2-4^

The study published in this issue of the *Arquivos Brasileiros de Cardiologia*^5^ conducts an epidemiological assessment of CVD in Portuguese-speaking countries (PSC) from 1996 to 2016, being, in that context, unprecedented and relevant. Despite some limitations, always present in that type of study, the analysis has considerable merit and allows us to draw very important conclusions. That study assesses, from an innovative perspective, CVD in a set of countries scattered around the world, which share a common language and cultural base, but have totally distinct geographic locations. In that type of analysis, the impact of local aspects, such as sanitation structures, health policies, economic and political conditions, on the parameters assessed must be properly considered, and that study does it in a very elegant way. The authors clearly indicate that the relative importance of the burden of CVD differs in the different PSC, and they directly relate those differences to the socioeconomic conditions of the countries. Of the CVD, ischemic heart disease is the major cause of death in all PSC, except for Mozambique and Sao Tome and Principe. In addition, the authors report that the most relevant risk factors for CVD, arterial hypertension and dietary factors, are common in the PSC. Furthermore, they conclude that "Genetic factors, implicit in the cultural identity, the factors inherent in the host, as well as the huge social inequality might have contributed to explain the mortality rates observed." It is worth noting that the authors report the general reduction in mortality from CVD as a common denominator among all the PSC, although the intensity of that reduction differs in the countries.

The introduction of several therapeutic strategies, such as drugs and medical devices, has determined a substantial reduction in mortality from CVD in general. In fact, the therapeutic and diagnostic advances in the cardiovascular field have contributed to an 80% increase in the life expectancy of the world population. That is an exceptional accomplishment. However, it is currently known that concomitantly with the decrease in mortality, several risk factors account for the increase in the prevalence of CVD. Arterial hypertension, diabetes, dyslipidemia, obesity and smoking habit have contributed to a general increase in the prevalence of CVD. It is worth noting that, despite the significant therapeutic advances, preventive measures, mainly the control of risk factors and promotion of healthy lifestyles, must be taken. Currently there is scientific evidence of the relationship between the implementation of preventive strategies and the corresponding reduction in cardiovascular events and mortality.^6,7^ An example is the immediate impact of the enforcement of the smoke-free environment legislation on the incidence of acute myocardial infarction.^8-10^ The reduction in hospitalization rates has been accompanied by a significant reduction in mortality rates^5^ in the acute phase and during follow-up, reflecting the disseminated use of evidence-based treatments, such as reperfusion therapies and drugs to prevent the progression of ischemic heart disease. Some of those interventions protect against other manifestations of CVD, such as stroke.

The study this editorial refers to confirms those aspects and emphasizes the need to develop preventive medicine policies, which have clearly shown great efficacy when properly implemented. In addition, it portrays, for the first time, a vast and robust set of data from countries that share several similarities, despite their specific features. The study should be properly disclosed to the sanitary authorities of the PSC to reinforce the need for measures to reduce the impact of CVD on those countries. Above all, it is an excellent example of cooperation that should be duly emphasized and replicated. I congratulate the authors and the Portuguese-speaking cardiology community.
